# Lewis-acid induced mechanochemical degradation of polyvinylidene fluoride: transformation into valuable products

**DOI:** 10.1039/d5sc05783c

**Published:** 2025-09-09

**Authors:** Minh Bui, Christian Heinekamp, Emil Fuhry, Steffen Weidner, Jörg Radnik, Mike Ahrens, Kerstin Scheurell, Kannan Balasubramanian, Franziska Emmerling, Thomas Braun

**Affiliations:** a Department of Chemistry, Humboldt Universität zu Berlin Brook-Taylor Str. 2 12489 Berlin Germany thomas.braun@cms.hu-berlin.de; b Federal Institute for Materials Research and Testing Richard-Willstätter Str. 11 12489 Berlin Germany; c School of Analytical Sciences Adlershof (SALSA) & IRIS Adlershof Albert-Einstein Str. 11 12489 Berlin Germany

## Abstract

Polyvinylidene fluoride (–[CH_2_CF_2_]_*n*_–, PVDF) waste poses significant environmental challenges due to its recalcitrant nature and widespread use. This study addresses the end-of-life management of PVDF by introducing a novel, sustainable mechanochemical approach for its valorisation. We investigated the degradation of PVDF into value-added materials using ball milling with anhydrous AlCl_3_ to achieve a quantitative mineralisation producing AlF_3_ and halide-functionalised graphite, along with gaseous products (HCl and CH_4_). Mechanistic key steps involve Lewis-acid catalysed C–F bond activation, dehydrofluorination and aromatisation. This approach provides an effective solution for PVDF waste management while offering a promising route for the production of high-value materials from polymer waste streams. Our findings contribute to sustainable practices in polymer recycling and resource recovery, respond to pressing environmental concerns associated with fluoropolymer disposal, and demonstrate the potential to convert polymer wastes into useful products.

## Abstract 2

Lewis acidic AlCl_3_ degrades mechanochemically PDVF *via* dehydrohalogenation and Scholl reactions, forming AlF_3_, fluorinated/chlorinated graphite, HCl and CH_4_.

## Graphical abstract

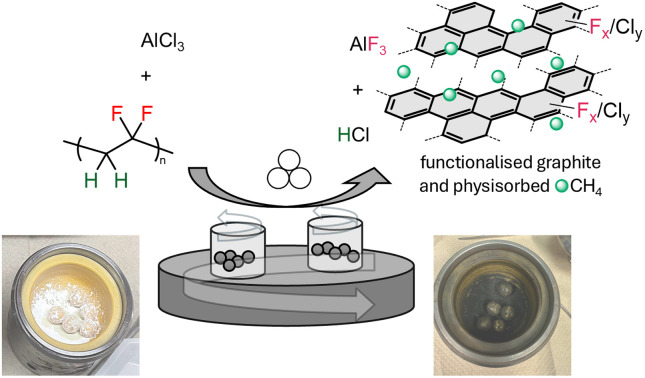

## Introduction

Polyvinylidene fluoride (–[CH_2_CF_2_]_*n*_–, PVDF) belongs to the class of perfluoro and polyfluoro alkyl substances (PFAS) and has been used extensively in chemical processing equipment, electronic components, water treatment systems and architectural coatings.^1,2^ PVDF acts as a vital binder in lithium-ion batteries to secure the cohesion between active materials and current collectors.^3^ While PVDF's exceptional chemical stability enhances battery performance, the increasing demand for these batteries translates to a growing challenge in waste management. Unlike many polymers, PVDF is highly resistant to degradation due to the inherent strength of its C–F bonds.^4–6^ Research efforts focused on developing efficient PVDF recycling methods are important for the sustainable development of lithium-ion battery technology, as they address the environmental impact of this key component in battery electrodes. Complementing these efforts, research into the controlled degradation of PVDF offers promising alternative strategies for responsible end-of-life management, potentially opening new avenues for material recovery and reuse.

Studies by Hori and co-workers demonstrated a complete mineralisation of PVDF in supercritical water at 380 °C using a 5.8-fold molar excess of oxygen.^7^ Interestingly, complete mineralisation was also achieved in subcritical water at 300 °C using a 31-fold molar excess of hydrogen peroxide relative to the fluorine content of PVDF.^8^ In both cases a carbon rich residue was formed which was not characterised further. Using molten NaOH, Yanagihara and Katoh accomplished the degradation of various PFAS including PVDF to NaF and carbonates.^9^ In addition, Baron and co-workers reported the degradation of PVDF performing mechanochemistry, in which NaOH was used as co-milling agent to form NaF.^10^ For the latter, water production causes strong agglomeration, which hampers an efficient fluoride/hydroxide exchange and again only unidentified carbon rich compounds and organic phases were generated.

In recent years, mechanochemistry^11^ has attracted significant interest because of its alignment with green chemistry principles by enabling solvent-free reactions, reducing waste, improving energy efficiency and developing new synthetic routes. This approach minimises environmental impact and maximises atom economy in chemical processes.^12,13^ For instance, Gouverneur and co-workers established a mechanochemical fluorination approach, using CaF_2_ to form fluorochemicals without prior production of HF.^14^ A review article by Aydonat *et al.* examines how mechanochemistry can enhance polymer sustainability.^15^ The paper discusses recent advances in mechanophores and applications to various polymers, contributing to efforts in sustainable materials development and plastic waste management.

Dehydrofluorination reactions using solid Lewis acidic Al compounds were reported in the literature. Aluminium chlorofluoride (ACF, AlCl_*x*_F_3−*x*_; *x*: 0.05–0.3) and tempered γ-Al_2_O_3_ at 700 °C have shown reactivity towards fluorinated molecules.^16–20^

**Scheme 1:**
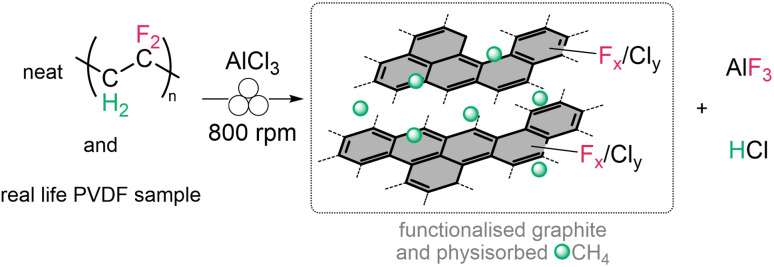
Reaction of PVDF and AlCl_3_ forming functionalised graphite, methane, aluminium fluoride and hydrogen chloride.

In this work, we report on a unique procedure for the quantitative mineralisation of PVDF by ball milling in the presence of AlCl_3_ to form AlF_3_ and, remarkably, a fluorinated and chlorinated graphite. Additionally, valuable gases such as HCl and CH_4_ were generated during the mechanochemical process (Scheme 1).

## Results and discussion

Mechanochemical degradation of PVDF was conducted in a Fritsch Premium Line 7 planetary mill from Fritsch GmbH, Germany. Anhydrous AlCl_3_ (267 mg, 2 mmol) and PVDF (192 mg, 3 mmol) were placed in a 45 mL ZrO_2_ jar equipped with a gassing lid and five ZrO_2_ balls (each 2.5 mg, 10 mm in diameter). After milling at 800 rpm for 7 h, an insoluble black powder was obtained. Common protic and aprotic solvents (H_2_O, EtOH, ^i^PrOH, DMSO, CH_2_Cl_2_, CHCl_3_, CCl_4_, CH_3_CN, acetone, C_6_H_6_) did not dissolve the black powder and impeded subsequent extraction attempts. Consequently, the bulk material was analysed as such; it is referred to as PMP*X* (planetary milled powder, *X* means the milling time in hours, PMP*X*m relates to the milled mixture using a PVDF membrane).

### Gas phase analytics

**Fig. 1:**
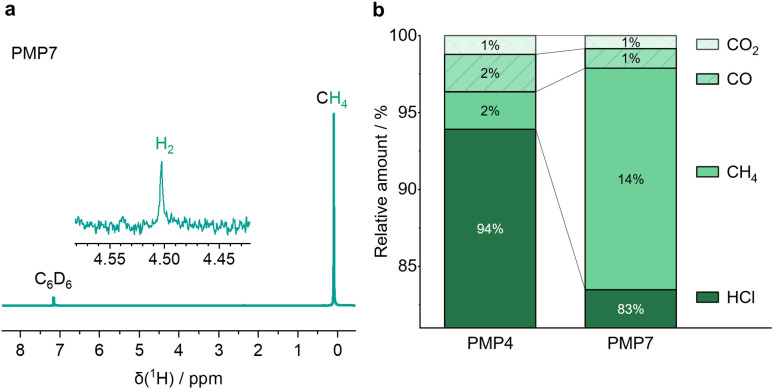
(a) ^1^H NMR spectrum (300 MHz, C_6_D_6_) from the gas phase after milling AlCl_3_ and PVDF for 7 hours (PMP7). (b) Results of the Optical Feedback Cavity Enhanced Absorption Spectroscopy (OFCEAS) for the gaseous contents of PMP4 and PMP7 using H_2_ as matrix gas.

The gaseous contents of the jars after milling were vacuum transferred into a C_6_D_6_ solution in JYoung NMR tubes for further characterisation. In the ^1^H NMR spectrum (Fig. 1a) a distinct signal at 0.10 ppm and a low intensity signal at 4.51 ppm were assigned to CH_4_ and H_2_, respectively. Furthermore, gas chromatography (SI Fig. 1) and OFCEAS (Optical Feedback Cavity Enhanced Absorption Spectroscopy) confirm the presence of CH_4_ after milling (Fig. 1b). In addition, OFCEAS experiments revealed the formation of HCl and trace amounts of CO and CO_2_, most likely originating from the polymer's oxidation during milling by residual air in the jar. Note that imperfect jar and gassing lid seals may allow some air ingress.

### X-ray powder diffraction, XRD

**Fig. 2:**
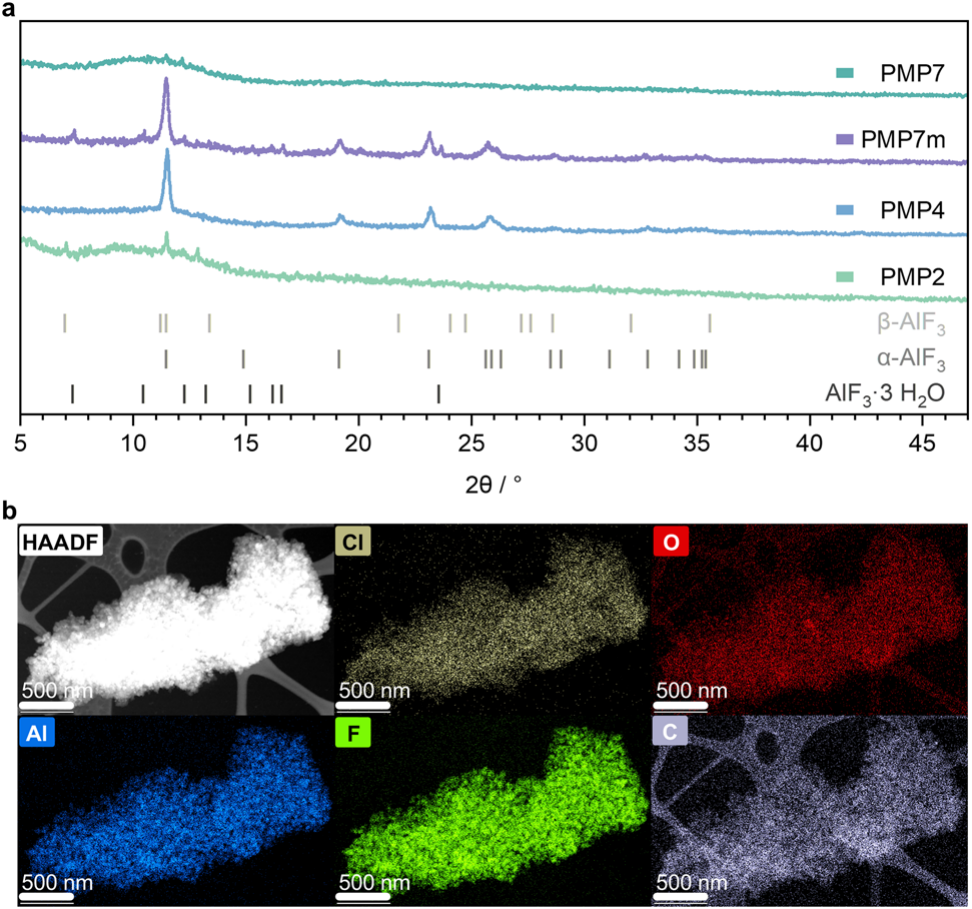
(a) X-ray powder diffractograms (Mo Kα source *λ* = 0.7107 Å) from milling approaches of AlCl_3_ and PVDF at various milling times. PMP*X* (planetary milled powder, *X* means the milling time in hours). For PMP7m PVDF membrane was used. The reference reflections for AlF_3_·3H_2_O, α- and β-AlF_3_ are depicted in grey. (b) Elemental mapping of PMP7 using STEM and EDX with a scale bar of 500 nm.

XRD patterns were collected from four samples, each consisting of AlCl_3_ and PVDF (using powdery PVDF or a PVDF membrane) mixtures that were milled for varying durations. The powders were measured without any further processing. Fig. 2a shows the influence of the milling time on the crystallinity. After 2 h of milling, weak reflections attributed to α-AlF_3_ and β-AlF_3_ were observable in the diffractogram for PMP2. After 4 h of milling the reflections for β-AlF_3_ diminished, while those for the thermodynamically stable α-AlF_3_ phase^21^ increased significantly, suggesting enhanced crystallinity of the α-AlF_3_ phase in PMP4. Notably, the diffraction pattern of the sample milled for 7 h displayed no distinct reflections. This result suggests a substantial amorphisation of the sample PMP7, caused by mechanical impact and increased defect concentrations due to prolonged milling.^22,23^ When AlCl_3_ and a PVDF membrane (PMP7m) was milled for 7 h, intense reflections attributed to α-AlF_3_ and minor reflections for AlF_3_·3H_2_O could be observed. Note that Scholz and colleagues reported on the mechanochemical generation of nanocrystalline aluminium hydroxide fluoride samples AlF_*x*_(OH)_3−*x*_·*n*H_2_O (*x* = 1.5) exhibiting pyrochlore structure by milling aluminium hydroxide and hydrous aluminium fluoride.^24^

### Scanning and transmission electron microscopy, S/TEM

S/TEM measurements were conducted to gain insight into the material. For this, vacuum was applied to PMP7 to remove HCl and CH_4_ from the material. Energy dispersive X-ray spectroscopy (EDX) suggests an atomic ratio of 1 to 3.1 for Al and F, which is in accordance with the formation of AlF_3_. Furthermore, EDX analysis revealed a slight excess of fluorine and chlorine atoms when accounting for the stoichiometry of AlF_3_. This is in accordance with the presence of a fluorinated and chlorinated material. Furthermore, the elemental mapping in Fig. 2b shows a homogenous distribution of Al, Cl, F and C atoms over the whole particles, which demonstrates that there is no phase separation of AlF_3_ and the graphitic material.

### Multinuclear MAS NMR spectroscopy

The ^27^Al MAS NMR spectrum of PMP7 displays only a signal at −16 ppm for [AlF_6_] entities (SI Fig. 15).^25^ The linewidth is 400–500 Hz less than the ones observed for other amorphous doped aluminium fluorides,^26,27^ suggesting less amorphicity.

**Fig. 3:**
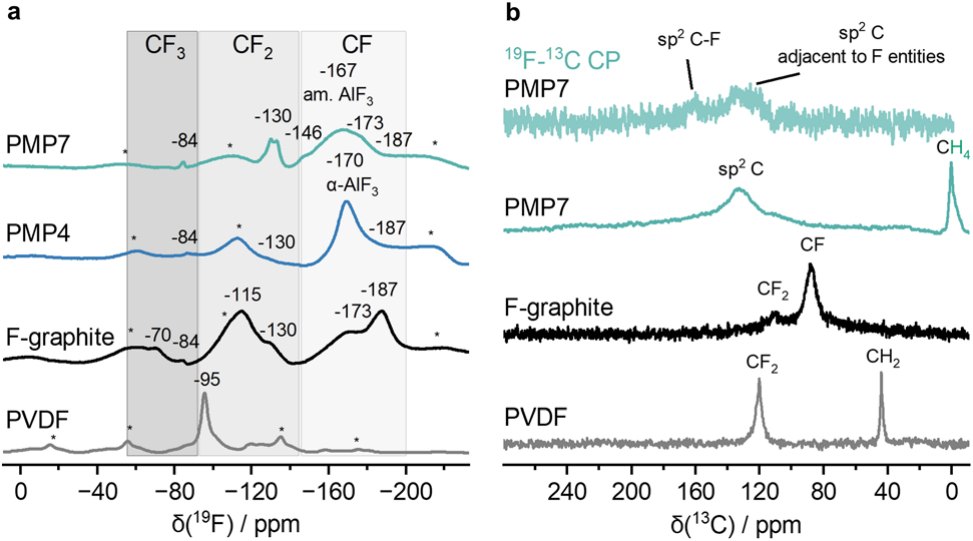
(a) ^19^F MAS NMR (*ν̃*_rot_ = 25 kHz) spectra of PMP4, PMP7, fluorinated graphite (F-graphite, >61 wt% F) and PVDF as reference; asterisks * represent spinning sidebands. am. stands for amorphous. (b) ^19^F–^13^C CP (top) and ^13^C{^1^H} MAS NMR (*ν̃*_rot_ = 10 kHz) spectra of PMP7, F-graphite and PVDF.

In the ^19^F MAS NMR spectrum (Fig. 3a) for PMP7 one can detect the signal for amorphous AlF_3_ moieties at −167 ppm.^25^ In addition, several signals were observed in the characteristic region for CF_*y*_ (*y* = 1, 2 or 3) moieties.^28^ The positions of these signals are consistent with those typically found in commercially available fluorinated graphite (also shown in Fig. 3a for comparison), which indicates the generation of functionalised graphite through the mechanochemical treatment. However, there are subtle differences. The signal at −130 ppm is more intense for PMP7, while the signal at −115 ppm observed in the commercial fluorinated graphite sample is missing for PMP7. The ^19^F MAS NMR spectrum of PMP4 shows a simplified profile, dominated by a prominent signal attributed to α-AlF_3_ at −170 ppm. Smaller, less intense signals are observed at −130 and −187 ppm. The comparison of the spectra suggests that longer milling times lead to an increased amount of CF_*y*_ (with *y* = 1, 2, 3) moieties. Note that the signal for PVDF at −95 ppm is not observed in the ^19^F MAS NMR for PMP4 and PMP7. The ^19^F MAS NMR spectrum of PMP7m is compatible with the presence of amorphous as well as α-AlF_3_ (SI Fig. 17).

The ^13^C{^1^H} MAS NMR spectrum of PMP7 depicted in Fig. 3b reveals a signal at 0 ppm at a characteristic chemical shift for CH_4_.^29^ The signal disappeared when vacuum was applied to the sample implying that the methane molecules were weakly bound to or physically trapped within the graphitic network. In addition, a broad signal centered at 130 ppm indicates the presence of sp^2^ carbon atoms. The ^19^F–^13^C CP MAS NMR spectrum depicted in Fig. 3a (top) reveals that some of these sp^2^ carbon atoms have a weak interaction with fluorine atoms within the graphitic material.^30^ Notably, a second signal at 160 ppm can be assigned to sp^2^ C–F moieties.^31^ Comparison with the ^13^C{^1^H} MAS NMR spectrum of fluorinated graphite revealed significant differences. The spectrum of fluorinated graphite only displayed signals at 88 and 110 ppm, corresponding to CF and CF_2_ functionalities,^32^ respectively. In contrast, the ^13^C{^1^H} MAS NMR spectrum of PMP7 is dominated by the broad sp^2^ C resonance at 130 ppm. The absence of signals at 88 and 110 ppm shows that PMP7 has significantly fewer CF and CF_2_ moieties than fluorinated graphite. This observation aligns with the suggested composition of PMP7, where sp^2^ C atoms are the primary component, with only a limited presence of C–F bonds. The ^13^C{^1^H} MAS NMR spectrum of PMP7m shows mainly a broad signal at −130 ppm (SI Fig. 18).

### Attenuated total reflection infrared spectroscopy, ATR-IR

**Fig. 4:**
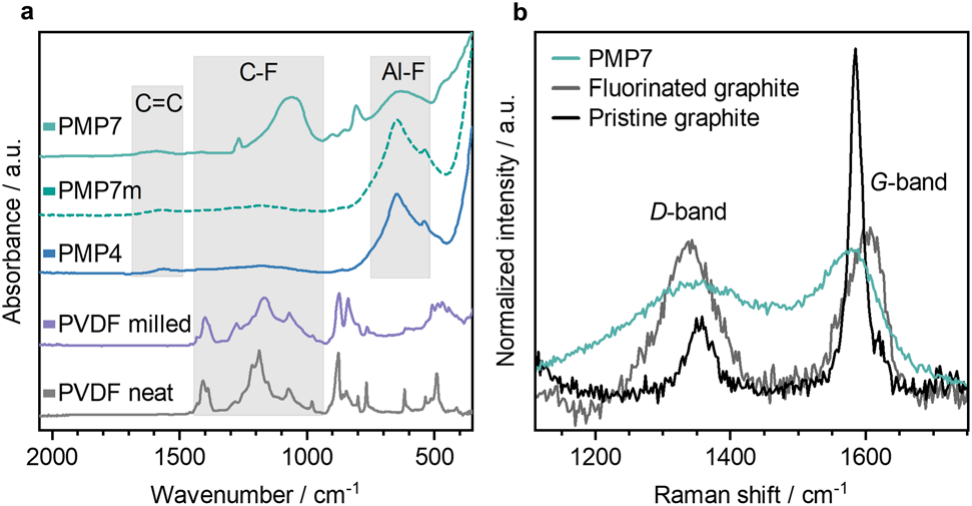
(a) ATR IR spectra (diamond) of PMP samples after 7 and 4 h of milling, PMP7 sample using PVDF membrane (PMP7m), 7 h milled PVDF and neat PVDF. (b) Raman spectra recorded at 532 nm for PMP7, fluorinated graphite (>61 wt% F) and pristine graphite.

ATR IR spectra (Fig. 4a) were measured to investigate the mechanochemical influence of ball milling on PVDF powder and PVDF membrane with and without the presence of AlCl_3_. In the IR spectra of PMP4, PMP7 and PMP7m a band at 640 cm^−1^ was observed and is assigned to Al–F vibrational bands.^33^ The broader linewidth of the Al–F band in PMP7 suggests an increased structural disorder or potential structural changes.^25–27^ In addition, the IR spectra of PMP4 and PMP7m show similarities to the spectrum of pure α-AlF_3_ (SI Fig. 23), with additional bands characteristic of C–F stretching vibrations at 1400–1000 cm^−1^.^34,35^ An extension of the milling time to 7 h of the PVDF powder results in intensified bands in this region. For PMP7 a distinct vibrational mode can be found at 805 cm^−1^ in a characteristic region for C–Cl bands suggesting that the graphite is also partially chlorinated during the milling process.^36^ Interestingly, the IR spectra of PMP7m and PMP4 exhibit notable similarities, while PMP7m and PMP7 show significant divergence. This suggests that when the PVDF membrane is used in the milling process, the resulting material PMP7m resembles PMP4 rather than PMP7. Additives of the real life PVDF sample may cause the difference of the degradation process. The formation of a graphitic structure by dehydrofluorination is indicated by a C=C band at 1590 cm^−1^.^37^ For comparison, after 7 h of milling neat PVDF, the IR data revealed a significant broadening of the C–F stretching bands between 1000 and 1400 cm^−1^, but no C=C bands that are characteristic for dehydrofluorination of PVDF were visible.^34,35^ Note that pyrolysis of PVDF at elevated temperatures has been reported to result in dehydrofluorination.^38^

### Raman spectroscopy

To gain a deeper understanding of the graphitic material, Raman spectra were recorded and compared with spectra of pristine graphite and fluorinated graphite (Fig. 4b). Graphite exhibits distinctive Raman features: the G-band (1582 cm^−1^), associated with the in-plane vibrations of sp^2^ carbons and the D-band (1354 cm^−1^), indicating defects or disorder.^39^ These bands were observed in all samples. However, the position and shape of these bands varied significantly between the samples. The milled bulk material PMP7 also shows similar D- and G-bands, but they are much broader than those in graphite. A broadening of D- and G-bands is typically associated with a high and variable degree of functionalisation and disorder in the graphitic material. This is also apparent from the Raman spectrum of commercial fluorinated graphite. The blue shift in the G-band (to 1590 cm^−1^) and the concomitant red shift (to 1339 cm^−1^) in the D-band for PMP7 are consistent with literature reports for functionalised graphite and are associated often with graphite amorphisation.^40^ Such shifts have been observed previously in oxidised or fluorinated graphite/graphene as well.^41,42^ In the case of PMP7, we attribute the functionalisation with fluorine and chlorine atoms as the primary reason for an altered electronic structure and bonding environment, which directly affects the C=C bond vibrational frequencies and contributes to the G- and D-band broadening and frequency shifts.

### X-ray photoelectron spectroscopy, XPS

**Fig. 5:**
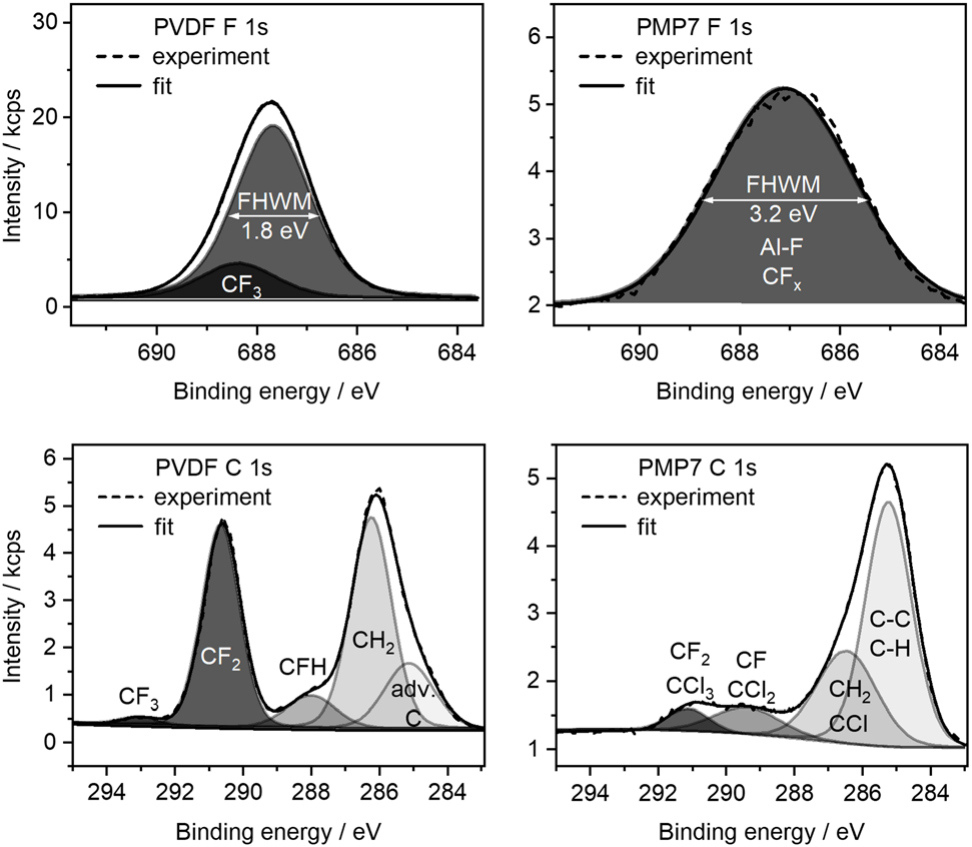
XPS high-resolution spectra of C 1s and F 1s of neat PVDF and PMP7. adv. C means adventitious carbon.

Raman spectroscopy indicated a distortion of the graphitic material, prompting further investigation. To elucidate the chemical state of PMP7, X-ray photoelectron spectra were measured, which is a surface-sensitive technique for near-surface layers. F 1s and C 1s XPS spectra of PVDF and PMP7 are depicted in Fig. 5. The F 1s XPS spectrum of PVDF predominantly exhibits a peak at 687.6 eV, characteristic for CF_2_ groups,^43^ along with a less intense peak at 688.3 eV attributed to CF_3_ groups. The CF_3_ groups are likely originating from terminal CF_3_ moieties within PVDF.^44^ In contrast, the F 1s XPS spectrum of PMP7 reveals a broad peak centered at 687.1 eV, likely resulting from the overlap of signals arising from CF_*x*_ and Al–F species.^43,45^ This interpretation is further supported by the significantly broadened full width at half maximum (FWHM) of 3.2 eV, compared to the typical FWHM of 1.8 eV observed for narrower peaks in PVDF. The C 1s XPS spectrum of PVDF exhibits peaks corresponding to CH_2_ in the neighbourhood of CF_2_ at binding energies of 286.2 and 290.7 eV, respectively.^43^ Additionally, small peaks attributed to CHF and CF_3_ moieties were observed at 287.9 and 293.0 eV, respectively.^43^ After a 7 hour milling process of PVDF in the presence of AlCl_3_, a decrease in the CF_2_ signal intensity was noted in the C 1s spectrum of PMP7. Concomitantly, peaks at 286.4, 289.4, and 291.1 eV associated with CCl, CCl_2_, and CCl_3_ moieties were observed,^43^ although these peaks could also be assigned to CH_2_, CF, and CF_2_ entities, respectively. The presence of chlorinated and fluorinated functionalities in the graphitic structure is expected to result in a higher binding energy shift of the sp^2^ C peak in XPS spectra. A clear identification of a distinct sp^2^ C peak is not possible, because the expected π to π* transition peaks^46^ at around 291–293 eV cannot be clearly identified due to the CF_2_ and CCl_3_ peaks in this region for PMP7. From the C 1s XPS analysis of PMP7, we can assume an approximate functionalisation of 14% by fluorine and chlorine atoms within the graphitic material (SI Table 2). Overall, the XPS data are in accordance with a defluorination of PVDF by the milling process.

### Proposed mechanism

**Scheme 2:**
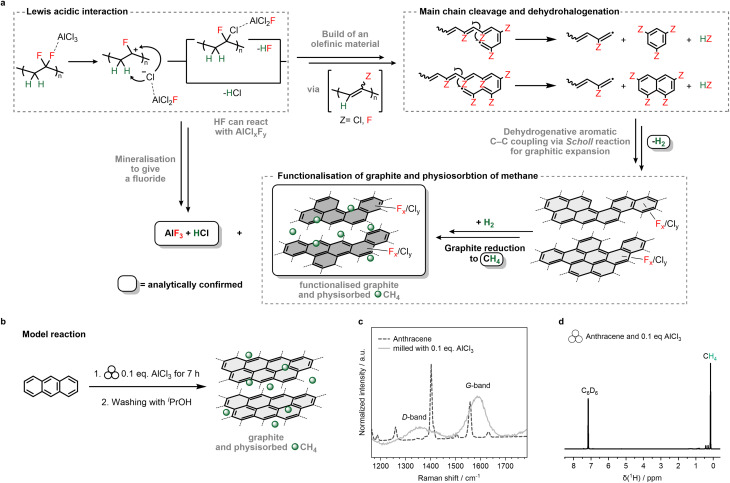
(a) Proposed reaction mechanism for the degradation of PVDF with AlCl_3_ using ball milling. (b) Model reaction of Scholl reaction using anthracene and 0.1 eq. AlCl_3_ forming graphite and methane. (c) Raman spectra of anthracene and product after milling anthracene and 0.1 eq. AlCl_3_ recorded at 532 nm. (d) ^1^H NMR spectrum (300 MHz, C_6_D_6_) from the gas phase after milling 0.1 eq. AlCl_3_ and anthracene.

The proposed PVDF degradation mechanism (Scheme 2a) involves a Lewis-acidic interaction of AlCl_3_ at a C–F bond of the –[CH_2_CF_2_]_*n*_– moiety producing a carbenium-like ion and an aluminate by fluorine–aluminium bond formation.^17,25,26,47,48^ In particular, the use of anhydrous AlCl_3_ as a Lewis-acidic co-milling agent is crucial for the reaction; hydrous AlCl_3_·6H_2_O or aluminium hydroxide/oxide proved to be ineffective. Subsequently, a chloride ion from the [AlCl_3_F]^**−**^ attacks the C–H bond, leading to the release of HCl gas by dehydrochlorination and eventually the formation of AlF_3_. Alternatively, the carbenium intermediate could undergo chlorination by the [AlCl_3_F]^**−**^ anion followed by Lewis-acid supported HF elimination (dehydrofluorination), leading to a partially chlorinated olefinic species. On the other hand, initial dehydrofluorination of fluorinated alkyl moieties in PVDF or derivatives can occur by release of HF molecules,^19,49,50^ which in turn can react further with AlCl_*x*_F_*y*_ (*x* = 3 − *y*, *y* ≠ 3) to generate AlF_3_ and HCl. Note that incomplete PVDF degradation was observed when less than two equivalents of AlCl_3_ were used. This is evidenced by the ATR-IR spectrum of the material obtained by a reaction of 1 eq. AlCl_3_ and 3 eq. PVDF (PVDF eq. are given with respect to the monomer block), where characteristic C–F vibrational modes remained detectable (SI Fig. 22). However, the resulting dehydrohalogenated product obtained with 2 eq. AlCl_3_ (a polyolefinic species) then undergoes C–C coupling and formation reactions to yield halogenated aromatic rings releasing again HF or HCl.^51^ To further elucidate the presence of intermediate radicals in the latter transformations, azobisisobutyronitrile (AIBN) was added to a mixture of AlCl_3_ and PVDF. Note that AIBN can be used for trapping alkenyl radicals.^52^ Milling of this mixture for 7 h yielded again an insoluble black powder. The gaseous products evolved were vacuum transferred into a C_6_D_6_ solution for subsequent gas and liquid phase analyses. NMR spectroscopic investigations and GC/MS data show the presence of 1,3,5-trifluorobenzene and chlorinated olefinic compounds (SI Fig. 2–8). In addition, MALDI-MS data revealed that the *M*_w_ of the material is 1000 *m*/*z* less when compared to *M*_w_ of PMP7 (2000 *m*/*z*; SI Fig. 20–21). This confirms that the addition of radicals hampers the formation of the graphitic network. High temperatures (up to 1000 K)^53–55^ during milling and AlCl_3_ as co-milling agent can facilitate the aromatic ring formation. Once aromatic rings are formed, a Lewis-acid-mediated *Scholl* reaction, involving H_2_ release, leads to the formation of fluorine- and chlorine-functionalised graphitic entities.^56,57^ The H_2_ evolution observed by the corresponding signals in the ^1^H NMR spectrum of the gaseous content from PMP7 depicted in Fig. 1a suggests that a reducing H_2_ atmosphere activates the graphitic material,^58,59^ promoting the formation of CH_4_, which adsorbs on the graphitic layers.

Importantly, the occurrence of the *Scholl* reaction and CH_4_ formation by H_2_ reduction steps are supported by the independent milling reaction of anthracene with a catalytic amount of 0.1 eq. AlCl_3_ (Scheme 2b), which produced graphitic entities (identified by Raman spectroscopy, Scheme 2c) and CH_4_ (detected by ^1^H NMR spectroscopy, Scheme 2d).

## Conclusions

In conclusion, this study highlights the potential of mechanochemistry to effectively transform fluorinated polymer waste such as PVDF into valuable products. The mechanochemical approach paves the way for further advancements in polymer degradation and demonstrates the transformative potential of mechanochemistry in opening up chemical synthesis and waste management towards a circular economy.^60^

Through ball milling with anhydrous AlCl_3_, a quantitative mineralisation of PVDF was achieved, yielding high-value materials such as AlF_3_ and functionalised graphite alongside gaseous HCl and CH_4_. AlF_3_ is used in various industries, including the production of aluminium, glass and ceramics.^61^ Functionalised graphite exhibits promising properties for applications in energy storage, catalysis and advanced materials.^62–64^ Note that a study by Ding *et al.* demonstrated that a AlF_3_ coated graphite anode exhibits a higher initial discharge capacity and improved rate performance.^65^ Traditional synthesis of fluorinated graphite typically involves reacting graphite with F_2_ or HF gas streams under high temperature and pressure conditions.^30,66^ In addition, the solvent-free approach in this study aligns with green chemistry principles.^12,13^

Whereas in general reaction mechanisms during mechanochemical treatment of PFAS remain largely unclear,^67^ the proposed mechanism for the PVDF degradation in this study by a Lewis-acid may contribute to an understanding of fundamental processes involved, and guide the development of efficient and sustainable degradation strategies. Model reactions have shown that *Scholl* reactions are a key-step for the generation of graphitic materials in polymer degradation. Thus, dehydrofluorination of PVDF is followed by the *Scholl* reaction that generates intermediate H_2_, which in turn implies that a partial oxidation of the carbon network occurs. H_2_ can react further to produce CH_4_. The presented strategy is, however, limited to fluoropolymers which can undergo dehydrofluorination.

Mechanochemical fluorinated polymer degradation is different from reported reductive methods. Thus, Crimmin and co-workers used a strong Mg(i) reducing agent to defluorinate polytetrafluoroethylene.^68^ The group of Qu and Kang developed a defluorination strategy of PFAS using a carbazole based photo reductant KQGZ (10,13-diphenyl-9*H*-dibenzo[*a*,*c*]carbazole) at low temperature.^69^

## Methods

Experimental procedures, characterisation methods and data for the control reactions can be found in the SI.

### General information and materials

All samples were prepared in an MBraun glovebox filled with argon or in JYoung NMR tubes using conventional Schlenk techniques. Anhydrous aluminium trichloride (purity 99.99%) was obtained from ABCR and used as received. Polyvinylidene fluoride powder was purchased from Apollo Scientific. The PVDF membrane (ROTI®Fluoro pore size 0.2 μm) was purchased from Carl Roth. Benzene-d_6_ was bought from Eurisotop and distilled over Solvona®. The reference materials graphite, fluorinated graphite (>61 wt% fluorine) and anthracene were obtained from Sigma Aldrich and used as received. Azobisisobutyronitrile (purity 98%) was obtained from Sigma Aldrich.

## Author contributions

M. Bui conceived the project, designed and carried out the experiments and wrote the manuscript. C. Heinekamp performed the S/TEM imaging and EDX analysis. E. Fuhry recorded the Raman spectra. S. Weidner performed the MALDI-TOF MS experiments. J. Radnik carried out the XPS measurements. MAS NMR experiments were measured by K. Scheurell. M. Ahrens, K. Balasubramanian, F. Emmerling and T. Braun supervised the project.

## Conflicts of interest

There are no conflicts to declare.

## Supplementary Material

SC-OLF-D5SC05783C-s001

## Data Availability

Details of the experimental procedures and characterization of the complexes can be found in the SI. Supplementary information is available. See DOI: https://doi.org/10.1039/d5sc05783c.
