# Supplementary material for: Lewis-acid induced mechanochemical degradation of polyvinylidene fluoride: transformation into valuable products
Source: Chem Sci. 2025 Sep 9;16(40):18903–10. doi: 10.1039/d5sc05783c (PMC12439207; doi:10.1039/d5sc05783c)
Supplement: SC-016-D5SC05783C-s001 [file SC-016-D5SC05783C-s001.pdf]

Supplementary Information (SI) for Chemical Science.
This journal is © The Royal Society of Chemistry 2025

Supplementary Information

Lewis-Acid Induced Mechanochemical Degradation of Polyvinylidene Fluoride: Transformation into Valuable Products

Minh Bui,[a] Christian Heinekamp,[a,b,c] Emil Fuhry,[c] Steffen Weidner,[b] Jörg Radnik,[b] Mike Ahrens,[a] Kerstin Scheurell,[a] Kannan Balasubramanian,[a,c,*] Franziska Emmerling,[a,b,*] and Thomas Braun[a,*]

[a] Department of Chemistry, Humboldt Universität zu Berlin, Brook-Taylor Str. 2, 12489 Berlin, Germany

[b] Federal Institute for Materials Research and Testing, Richard-Willstätter Str. 11, 12489 Berlin, Germany

[c] School of Analytical Sciences Adlershof (SALSA) & IRIS Adlershof, Albert-Einstein Str. 11, 12489 Berlin, Germany

Table of Contents

Mechanochemistry 2
Analytics for the gaseous content 2
Optical feedback cavity enhanced absorption spectroscopy (OFCEAS) 2
Gas chromatography 3
Gas chromatography and mass spectrometry 3
Powder X-ray reflection diffraction 6
Scanning / transmission electron microscopy 7
Liquid MAS NMR spectroscopy 8
Solid-state MAS NMR spectroscopy 9
MALDI TOF Mass spectrometry 12
Infrared spectroscopy 14
Raman spectroscopy 15
X-ray photoelectron spectroscopy 16

Mechanochemistry

Planetary milling was conducted in a Fritsch Premium Line 7 using 45 mL ZrO_2 jars equipped with a gassing lid, a Viton® O-ring and five ZrO_2 balls (each 2.5 mg, 10 mm diameter).

a) Anhydrous $AlCl_3$ (267 mg, 2 mmol) and PVDF or commercially available PVDF membrane (192 mg, 3 mmol; with respect to the monomer block) were placed into jars. After milling at a rotational speed of 800 rpm for 1x30 min, 12x20 min (4 h) or 21x20 min (7 h) with 5 min breaks in between, a black powder could be obtained. PVDF was also milled for 7 h with the same condition as PMP7. The commercially available PVDF membrane (ROTI®Fluoro pore size 0.2 μm) was milled for 4x30 min (2 h) before usage.

b) Anthracene (178 mg, 1 mmol) and anhydrous $AlCl_3$ (13 mg, 0.1 mmol) were placed into the jars. After milling at a rotational speed of 800 rpm for 21x20 min (7 h) with 5 min breaks in between, a greyish powder could be obtained. Then, the powder was washed with isopropanol (5x5 mL) to obtain a dark grey powder, which was used for Raman spectroscopy.

c) As a control reaction, a mixture of 2 eq. $AlCl_3$, 3 eq. PVDF (eq. are given with respect to the monomer block) and 1.5 eq. azobisisobutyronitrile (AIBN) (450 mg in total) was milled for 30 min and 21x20 min (7 h) to investigate the proposed radical mechanism in Scheme 2a. A black powder could be obtained.

Analytics for the gaseous content

After milling the substrates, the gaseous content from the jars were vacuum transferred into a JYoung NMR tube filled with 0.6 mL C_6D_6 at −196 °C. The solutions were used for NMR spectroscopic investigations or for GC/MS.

Optical feedback cavity enhanced absorption spectroscopy (OFCEAS)

Low pressure sampling optical feedback cavity enhanced absorption spectroscopy (OFCEAS) was conducted at a ProCeas AP2E analyser from Durag Group. The analyser had a 20 km laser path length and was operated at a reduced pressure of 0.1 bar in the inlet. The measuring cell was maintained at a constant temperature of 40 °C. A 100 μm nozzle was used to achieve a sampling flow rate of 250 mL_n/min with

H_2 as the matrix gas. The pipes and measuring cell were coated with SilcoNert® to minimise adsorption. An analyte specific laser was used to detect CO, CO_2, HCl and CH_4. The milling jars after reaction were directly connected to the OFCEAS device for the measurement.

Gas chromatography

GC measurements were conducted on a TRACE 1300 Series GC II instrument from S+H Analytics. A 30 m long TGBondQ+ column was used and is specifically designed for the separation of hydrocarbons, with hydrocarbons eluting later. The carrier gas for the GC was He 6.0 from Linde. The sample gas was H_2 6.0 also from Linde, with the sample gas being introduced into the carrier gas via diffusion through a mass flow controller. A backflush mechanism was used to prevent hydrogen from reaching the detector. For the GC measurement of the gaseous content the milling jars after reaction were directly connected to the GC device via the gassing lid.

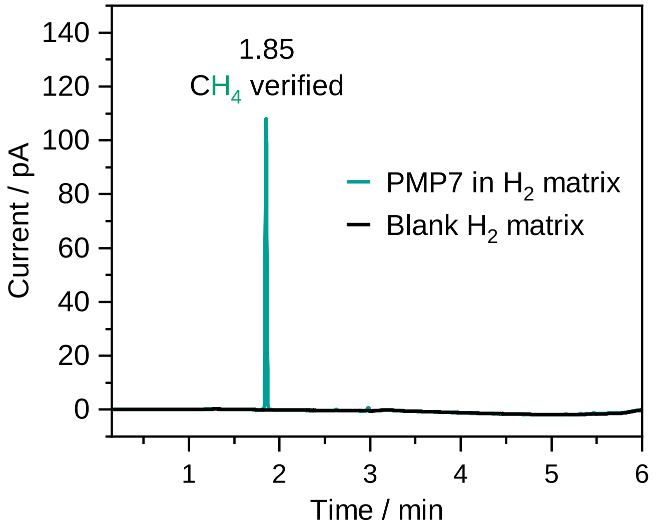

Supplementary Fig. 1. GC spectrum of the gaseous content of PMP7 in H_2 matrix.

Gas chromatography and mass spectrometry

GC/MS analysis was conducted on an AGILENT 7890B gas chromatograph (HP5 column, 30 m) with a flame-ionization detector coupled to an EI-MS AGILENT 5977B spectrometer with a triple-axis detector. The instrument was equipped with an autoinjector AGILENT G4513A (injection of approx. 10 µL). The GC method starts with an oven temperature of 25 °C (hold time 0.5 min) and includes two ramps (ramp 1: 25–

50 °C, 25 °C/min; ramp 2: 50–250 °C, 25 °C/min, each hold time 5 min), a total run-time of 29 min and a solvent delay of 2.9 min. MS peaks were analysed and compared with the library database of NIST MS Search 2.3. All EI-MS spectra of the detected peaks were in good agreement with the library database of the expected substances.

As a control reaction, a mixture of 2 eq. $AlCl_3$, 3 eq. PVDF (with respect to the monomer block) and 1.5 eq. AIBN was milled for 0.5 h within a 45 mL ZrO_2 jar equipped with 5 ZrO_2 balls. The gaseous content was vacuum transferred to a C_6D_6 solution and analysed by GC/MS. The data are shown below.

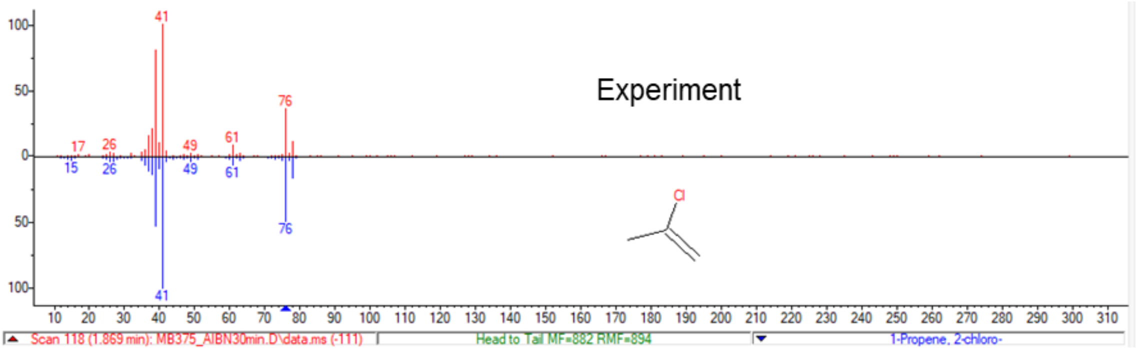

Supplementary Fig. 2. GC/MS analysis showing 2-chloro-prop-1-ene.

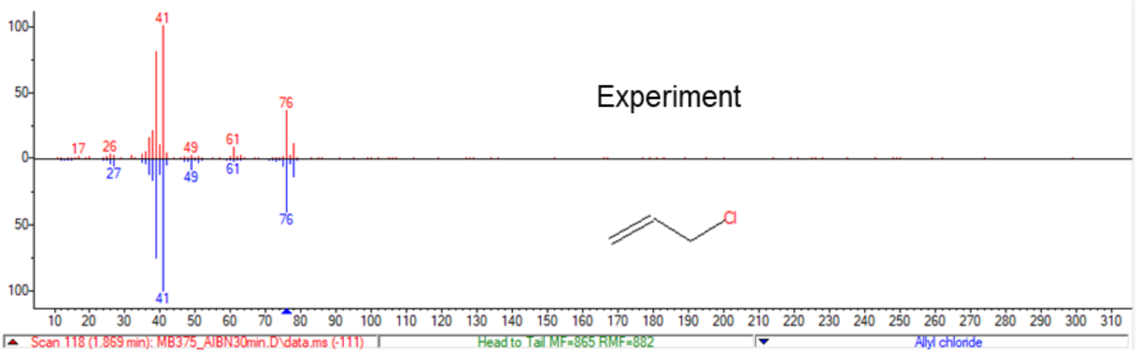

Supplementary Fig. 3. GC/MS analysis showing allyl chloride.

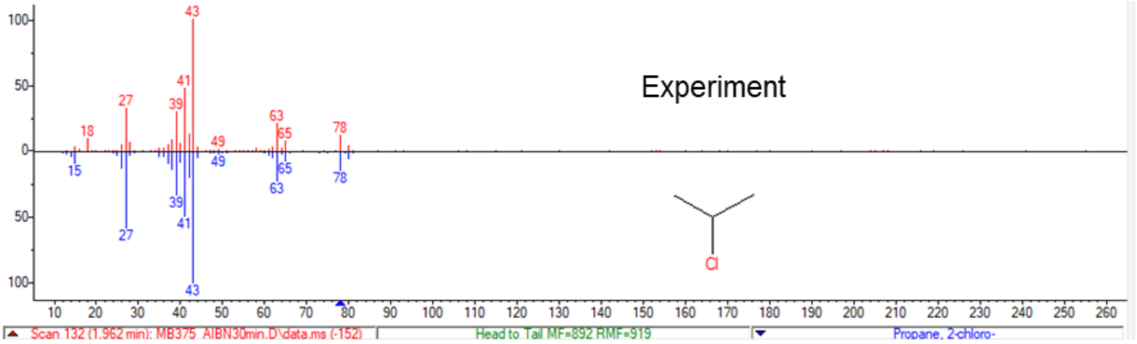

Supplementary Fig. 4. GC/MS analysis showing 2-chloropropane.

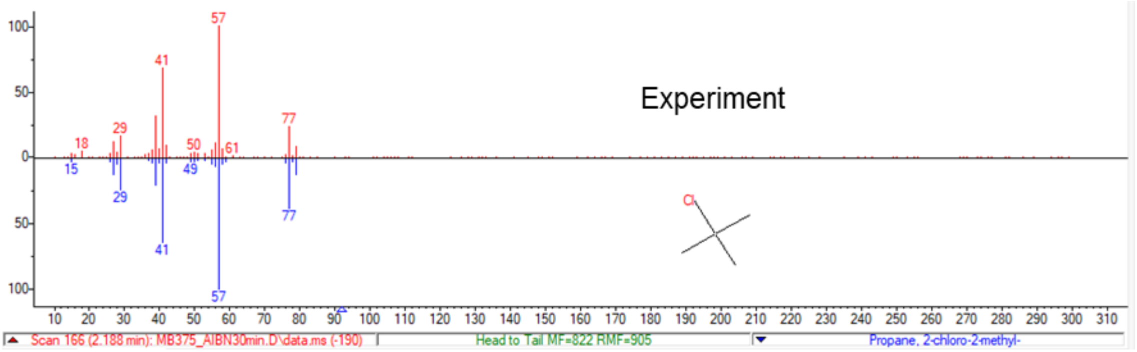

Supplementary Fig. 5. GC/MS analysis of showing 2-chloro-2-methylpropane.

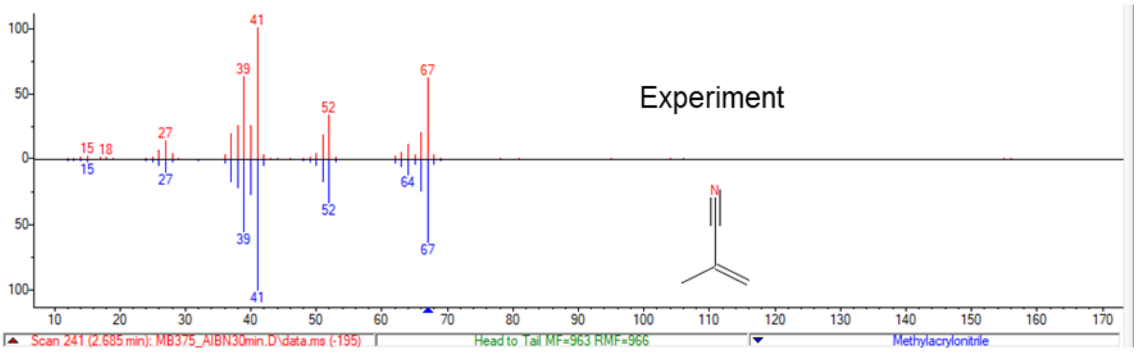

Supplementary Fig. 6. GC/MS analysis showing methylacrylonitrile.

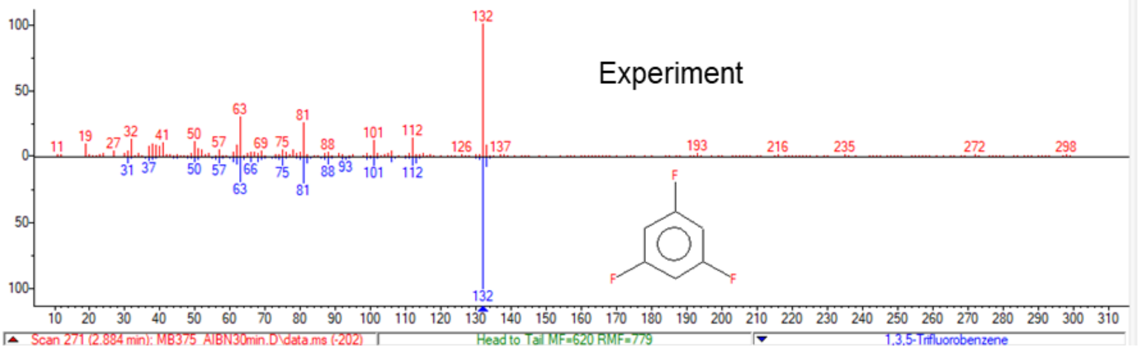

Supplementary Fig. 7. GC/MS analysis showing 1,3,5-trifluorobenzene.

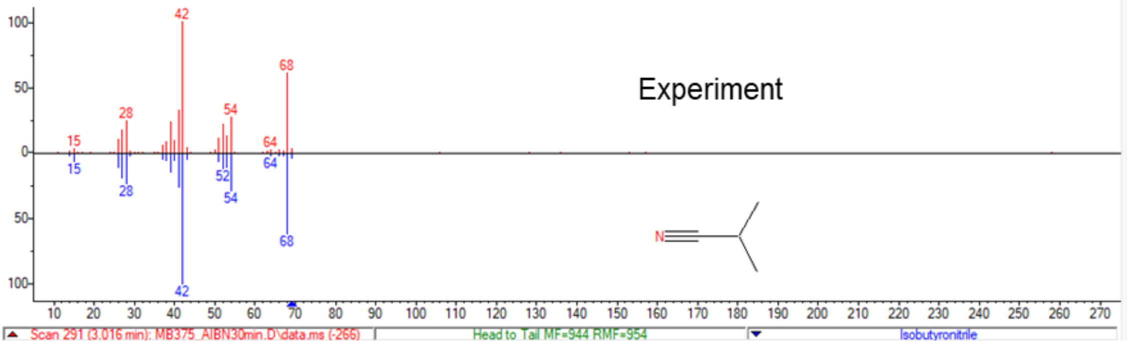

Supplementary Fig. 8. GC/MS analysis showing isobutyronitrile.

Powder X-ray reflection diffraction

X-ray powder diffraction data were collected on a STOE Stadi MP diffractometer equipped with a Dectris Mythen 1 K linear silicon strip detector and Ge(111)double-crystal monochromator (Mo Kα radiation with λ = 0.7107 Å) in a transmission geometry. The measurements were performed with a step size of 0.5 ° (2θ) with an integration time of 150 s per step over a range of 5-50 ° (2θ).

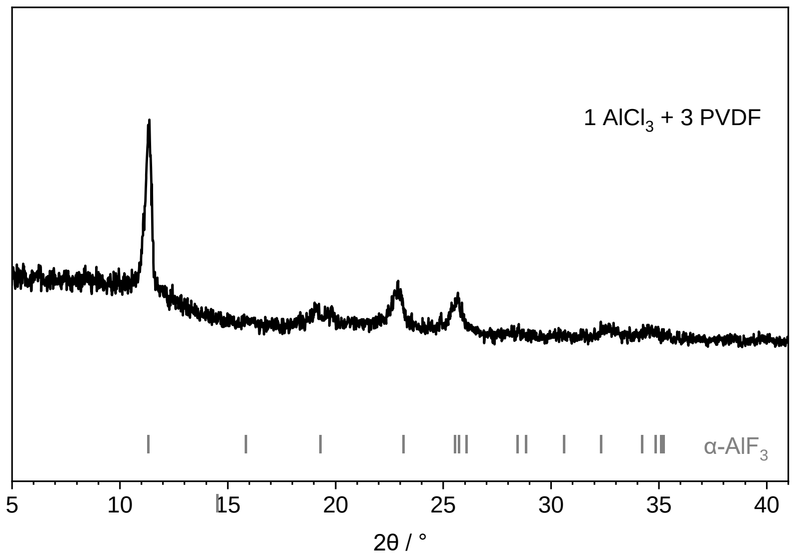

Supplementary Fig. 9. X-ray powder diffractograms (Mo Kα source λ = 0.7107 Å) from milling 1 eq. $AlCl_3$ and 3 eq. PVDF; with respect to the monomer block. The reference reflections for α-AlF_3 are depicted in grey.

Scanning / transmission electron microscopy

High-resolution transmission electron microscopy (HRTEM), high-angle annular dark-field scanning transmission electron microscopy (HAADF-STEM) and energy dispersive X-ray analysis (EDX) elemental mapping were carried out on a FEI Talos F200S scanning/transmission electron microscope (S/TEM) at an acceleration voltage of 200 kV. A dry TEM grid preparation was carried out. Therefore, TEM grids were carefully swiped across the powder samples. The excess of powder on the grids were removed by tapping lightly. The determination of the atomic fraction and atomic error by EDX analysis was conducted by averaging the data of five randomly selected spots on the particle.

Supplementary Tab. 1: EDX analysis of PMP7.

Element	Atomic fraction / %	Atomic error / %
C	30.61	3.03
O	5.63	1.27
F	51.74	5.22
Al	14.89	2.82
Cl	2.79	0.56

Using the EDX data and considering that AlF_3 is still present in the PMP7 material, we can estimate that the graphite contains approximately 7% fluorine. Please note that this is an imprecise estimation.

Liquid MAS NMR spectroscopy

Liquid NMR spectra were recorded at Bruker DPX 300, Bruker AVANCE II 300, or Bruker AVANCE II 500 spectrometers at room temperature using tetramethyl silane (TMS) as external standard. ^{1}H NMR chemical shifts δ were referenced to residual C_6D_5H (δ = 7.16 ppm). ^{19}F NMR spectra were calibrated externally to $CFCl_3$ (δ=0 ppm).

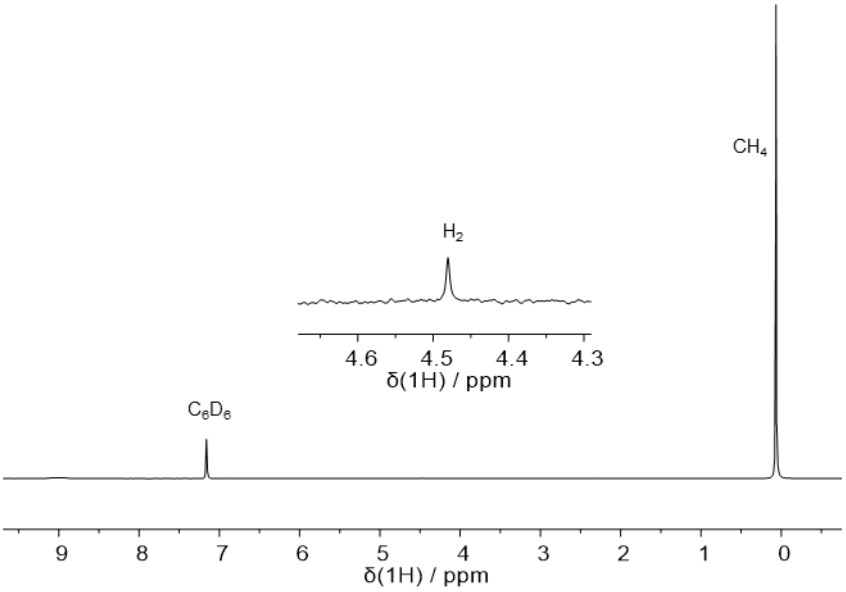

Supplementary Fig. 10. ^{1}H NMR (300 MHz, C_6D_6) spectrum of the gaseous content from PMP7m using PVDF membrane.

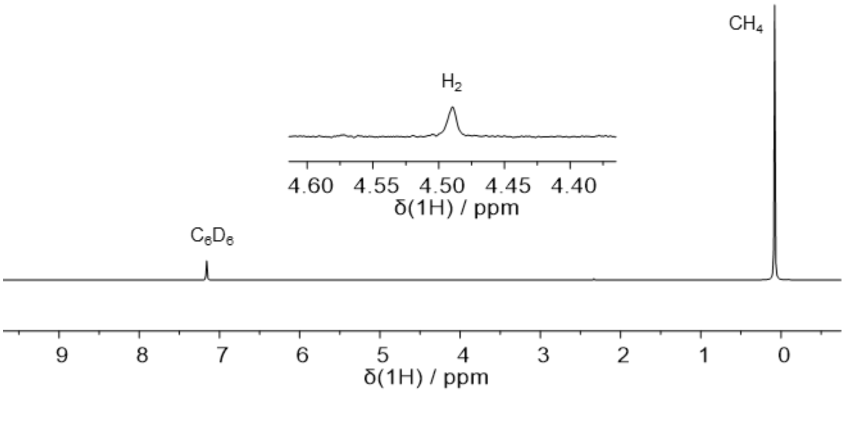

Supplementary Fig. 11. ^{1}H NMR (300 MHz, C_6D_6) spectrum of the gaseous content from milling reaction of 1 eq. $AlCl_3$ + 3 eq. PVDF (with respect to the monomer block) for 7h.

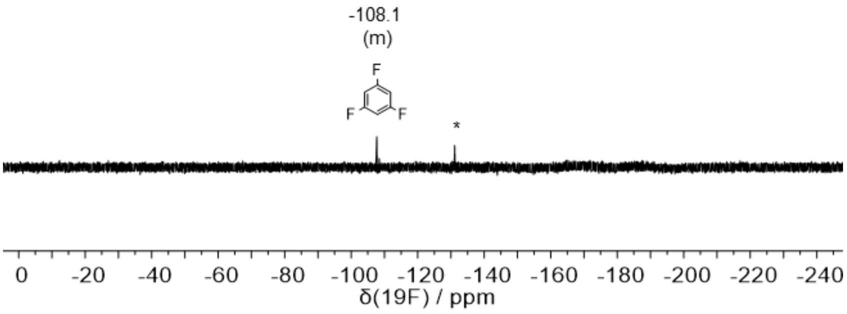

Supplementary Fig. 12. ^{19}F NMR (284 MHz, C_6D_6) spectrum of the gaseous content from PVDF, $AlCl_3$ and AIBN. Asterisk (*) stands for Si–F moiety. HF may react with the glass from the JYoung NMR tube.

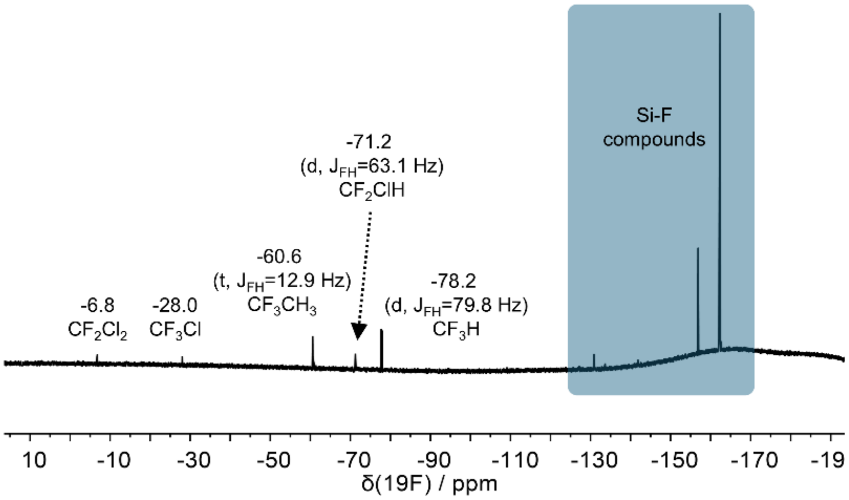

Supplementary Fig. 13. ^{19}F NMR (284 MHz, C_6D_6) spectrum of the gaseous content from milling reaction of 1 eq. $AlCl_3$ + 3 eq. PVDF (with respect to the monomer block) for 7h. HF may have reacted with the glass from the JYoung NMR tube to form Si–F compounds.

The reaction of 1 eq. $AlCl_3$ with 3 eq. PVDF (with respect to the monomer block) shows new signals in the ^{19}F NMR spectrum (SI Fig. 13), which were not present in the reaction using a 2:3 $AlCl_3$:PVDF (monomer block CH_2CF_2) ratio. These new signals likely originate from the formation of smaller chlorinated and fluorinated methane and ethane species. This suggests that the substoichiometric amounts of $AlCl_3$ in the 1:3 reaction are insufficient for full polymer breakdown, leading instead to these smaller, partially degraded fragments.

Solid-state MAS NMR spectroscopy

Solid-state MAS (magic angle spinning) nuclear magnetic resonance spectra were measured at a Bruker AVANCE 400 (B_0 = 9.4 T) spectrometer at room temperature.

^{1}H, ^{19}F and ^{27}Al MAS NMR spectra were measured on using 2.5 mm rotors at a rotation frequency of 25 or 20 kHz and a recycle delay of 5 s. ^{19}F-^{13}C-CP (cross polarisation) MAS NMR spectra were measured in 4 mm rotors at a rotation frequency of 10 kHz, a recycle delay of 3 s and a contact time of 10 ms. The proton decoupled ^{13}C MAS NMR spectra were performed at 10 kHz with a recycle delay of 20 ms. The chemical shifts were referenced to a $CFCl_3$ (δ = 0 ppm) standard for ^{19}F and a 1 M aqueous solution of $AlCl_3$ (δ = 0 ppm) for ^{27}Al. α-AlF_3 served as the external standard for both nuclei. Values of isotropic chemical shifts of ^{13}C are given with respect to $Si(CH_3)_4$ and are measured against adamantane as secondary standard.

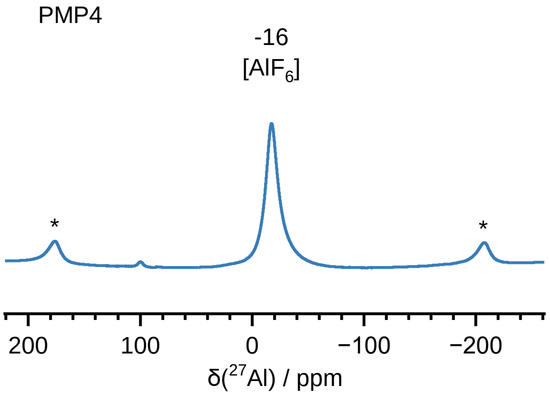

Supplementary Fig. 14. ^{27}Al MAS NMR ($\tilde{\nu}_{rot}$ = 20 kHz) spectrum of PMP4; asterisks * represent spinning sidebands.

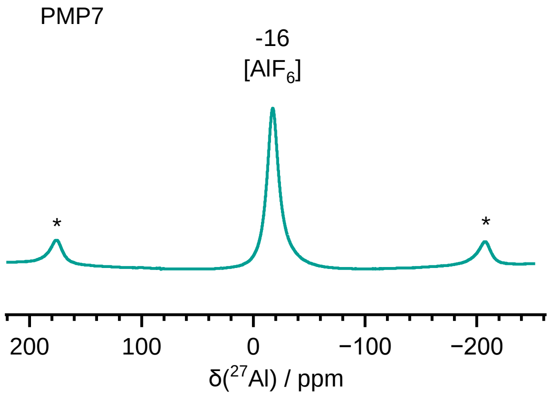

Supplementary Fig. 15. ^{27}Al MAS NMR ($\tilde{\nu}_{rot}$ = 20 kHz) spectrum of PMP7; asterisks * represent spinning sidebands.

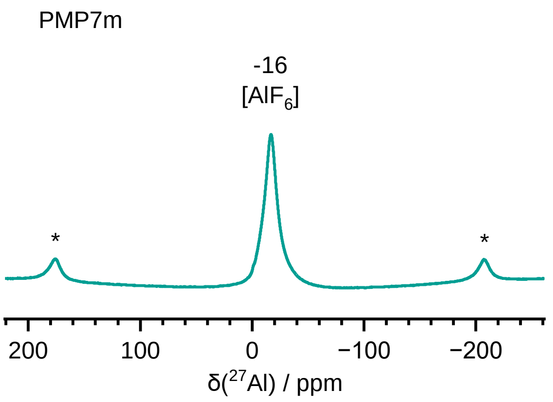

Supplementary Fig. 16. ^{27}Al MAS NMR ($\tilde{\nu}_{rot}$ = 20 kHz) spectrum of PMP7m; asterisks * represent spinning sidebands.

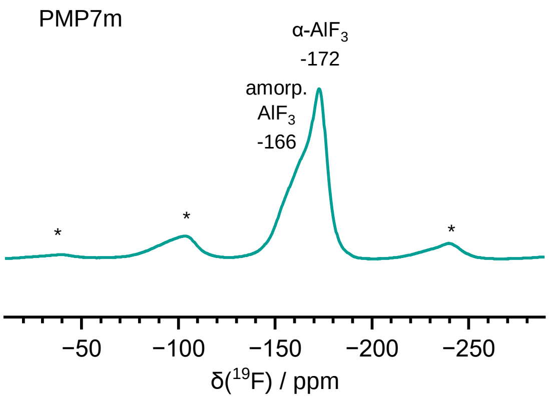

Supplementary Fig. 17. ^{19}F MAS NMR ($\tilde{\nu}_{rot}$ = 20 kHz) spectrum of PMP7m; asterisks * represent spinning sidebands.

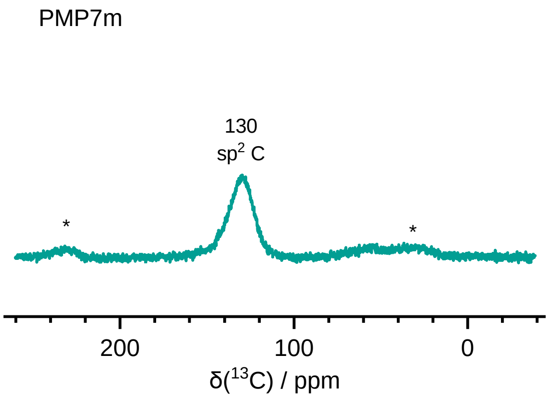

Supplementary Fig. 18. $^{13}C\{^{1}H\}$ MAS NMR ($\tilde{\nu}_{rot}$ = 10 kHz) spectrum of PMP7m after gas transfer; asterisks * represent spinning sidebands.

MALDI TOF Mass spectrometry

All experiments were performed on an Autoflex maX mass spectrometer from Bruker equipped with a 355 nm Nd-YAG laser and 2000 Hz repetition rate working in linear mode. Typically, 2000 shots recorded at 4 randomly chosen positions of the spot were accumulated for one spectrum. The reproducibility of the results was checked by measuring multiple sample spots. Data recording, calibration and evaluation was performed using the instrument software and Origin. A *trans*-2-[3-(4-tert-Butylphenyl)-2-methyl-2-propenyliden]malononitril (DCTB) matrix was used for PVDF, but no matrix was used for the PMPX samples.

To show the successful degradation of PVDF after milling with $AlCl_3$, MALDI-TOF spectra were recorded from neat PVDF, PMP7 and PMP7+AIBN (azobisisobutyronitrile). The mass spectrum of neat PVDF shows a typical pattern for a high polydisperse polymer (SI Fig. 19). At around 7800 Da one can detect peak spacing of 64 Da, which corresponds to the monomer block of PVDF. Furthermore, a broad band is centered at around 3400 Da with peak distances of 14 Da indicating [CH_2] fragments. These fragments might result from the laser incident causing PVDF degradation.[1] In the mass spectrum of PMP7 (SI Fig. 20) a very broad peak is observed at around 2000 Da with a regular spacing of 24 Da between peaks. This spacing suggests the presence of [C_2] fragments, which is characteristic for graphite.[2] No peaks with a spacing of 64 Da were detected in accordance with the degradation of PVDF by milling. As a control reaction, a mixture of 2 eq. $AlCl_3$, 3 eq. PVDF (with respect to the monomer block) and 1.5 eq. AIBN was milled for 7 h and an insoluble black powder could be obtained. LDI-TOF analysis of this powder (SI Fig. 21) revealed a shift of the M_w signal at around 2000 m/z to lower m/z values at around 1010 m/z. This observation suggests that the presence of radicals, generated from the decomposition of AIBN, effectively inhibits the formation of larger graphitic moieties within the degraded material.

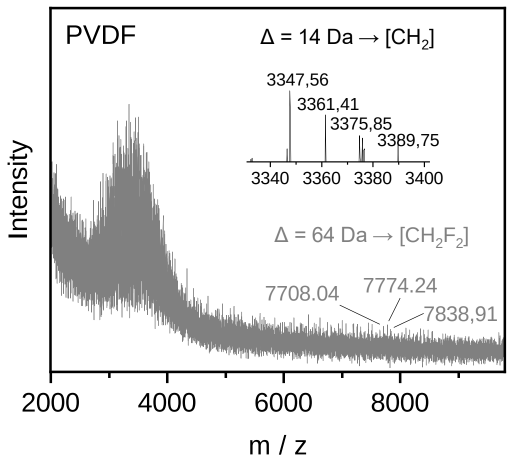

Supplementary Fig. 19. MALDI-TOF spectrum of neat PVDF in a trans-2-[3-(4-tert-Butylphenyl)-2-methyl-2-propenyliden]malononitril (DCTB) matrix.

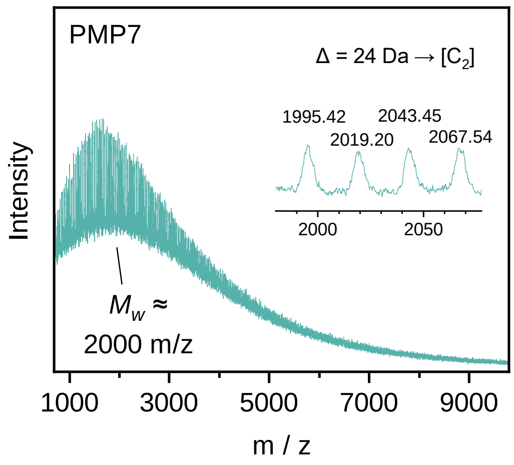

Supplementary Fig. 20. LDI-TOF spectrum of PMP7.

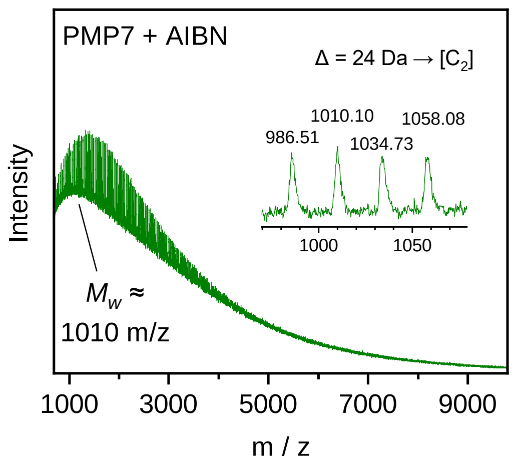

Supplementary Fig. 21. LDI-TOF spectrum of PMP7+AIBN.

Infrared spectroscopy

The IR spectra were recorded in an MBraun glovebox filled with argon at a Bruker Alpha II spectrometer with a diamond ATR (attenuated total reflectance) measuring unit (Pyroelectric DTGS detector).

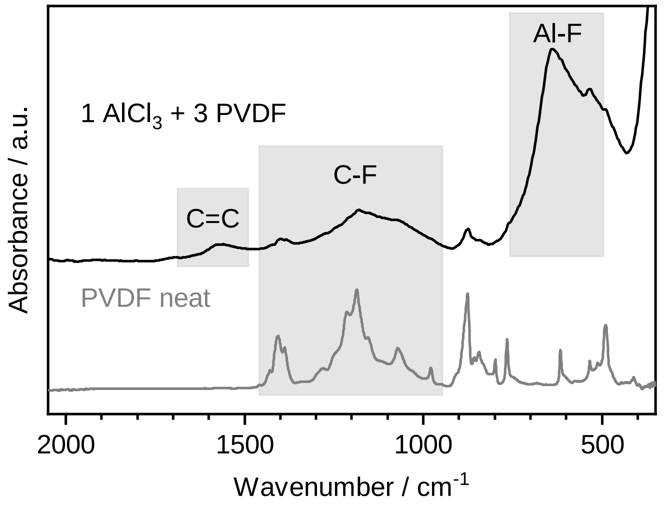

Supplementary Fig. 22. ATR IR spectrum of the bulk material from milling 1 eq. $AlCl_3$ + 3 eq. PVDF for 7h.

When mechanochemically treating 1 eq. of $AlCl_3$ with 3 eq. of PVDF (with respect to the monomer block), vibrational modes for Al–F at 660 cm^{-1} and C=C at 1600 cm^{-1} appear in the ATR IR spectrum (SI Figure 22), suggesting the formation of AlF_3 and graphitic entities. The presence of C–F vibrational modes associated with PVDF indicates an incomplete degradation of the polymer structure. This observation suggests that a stoichiometric ratio of $AlCl_3$ is crucial for achieving complete polymer decomposition. Specifically, 2 eq. of $AlCl_3$ are required to effectively degrade the polymer under these reaction conditions.

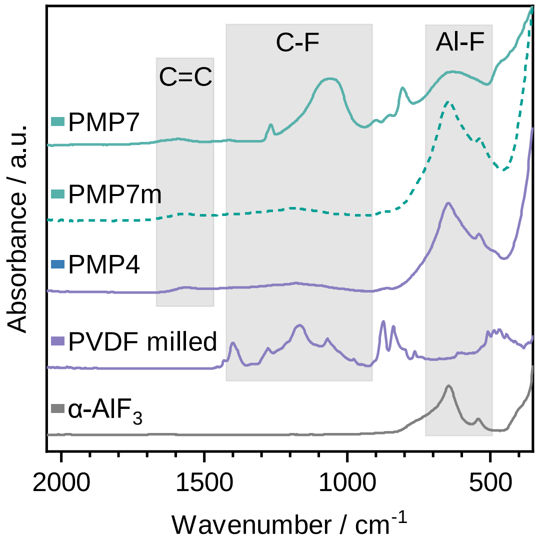

Supplementary Fig. 23. Comparison of the ATR IR spectra of PMP7, PMP7m approach using PVDF membrane, PMP4, PVDF milled and α-AlF_3.

Raman spectroscopy

Raman spectra were collected using a JASCO NRS-4100 Raman spectrometer. The spectrometer was equipped with a 1650 x 256 CCD detector (Andor; air/Peltier-cooled, operating temperature: −61 °C), a 900 L/mm grating, a diode laser with excitation of 532 nm, and a 100X (NA 0.90) objective. The laser power was maintained at 5.6 mW. Each Raman spectrum was recorded with an exposure time of 2 s and 3 accumulations.

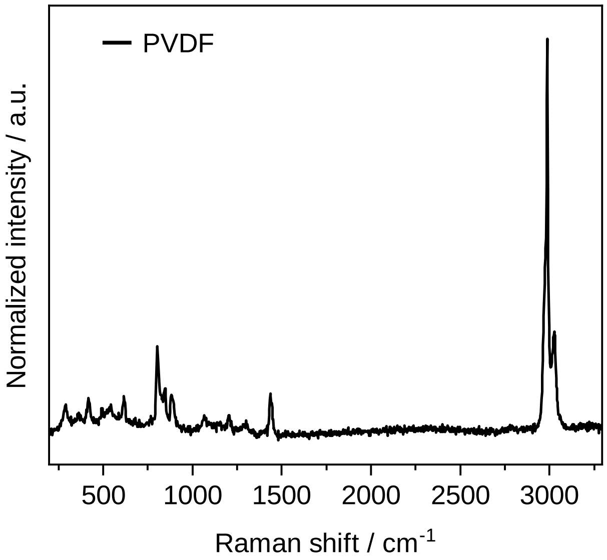

Supplementary Fig. 24: Raman spectrum of neat PVDF acquired at 532 nm.

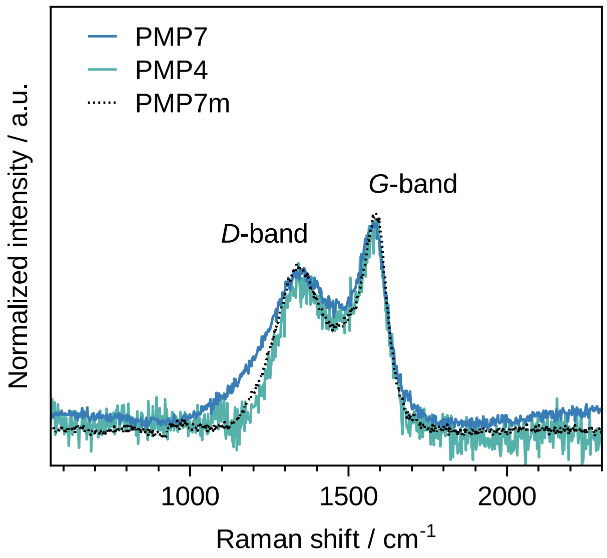

Supplementary Fig. 25: Comparison of the Raman spectra of PMP7, PMP7m and PMP4 acquired at 532 nm.

X-ray photoelectron spectroscopy

XPS analyses were conducted using the ULVAC-PHI "Quantes" spectrometer (Chanhassen, USA) with an Al Kα source (E = 1486.6 eV). The X-ray beam spot size was adjusted to 100 µm for these experiments. Photoelectrons were gathered at a 45 ° emission angle. The Al Kα source was positioned with its X-ray beam perpendicular to the sample surface. The vacuum within the sample chamber was maintained below 10^{-6} Pa throughout the experiments. Analyses were conducted on three distinct areas of each sample, utilizing low energy electrons and Ar^+ ions for charge neutralization. For the quantitative analysis, the survey spectra were used, which were measured with a pass energy of 280 eV and a step size of 1 eV. The binding energy (BE) scale was calibrated according to a PHI procedure that uses binding energy data from ISO 15472.[3] The intensity was calibrated with the PHI MultiPaK software with a method based on an idea of Seah.[4]

The percentage composition (in at-%) was determined from the peak areas after subtracting Shirley backgrounds and using relative sensitivity factors provided by the manufacturer with MultiPaK. PHI MultiPak Software Version 9.9.2 was used for the quantification of the atomic concentration using the automatic peak indexing routine. For the peak fitting with the Unifit software, the PHI datasets had to be converted from SPE to NPL format. For the peak fitting, Unifit 2025 (Unifit-Software, Leipzig, Germany) was employed using a sum of Gaussian-Lorentzian curves and a modified Tougaard background.[5]

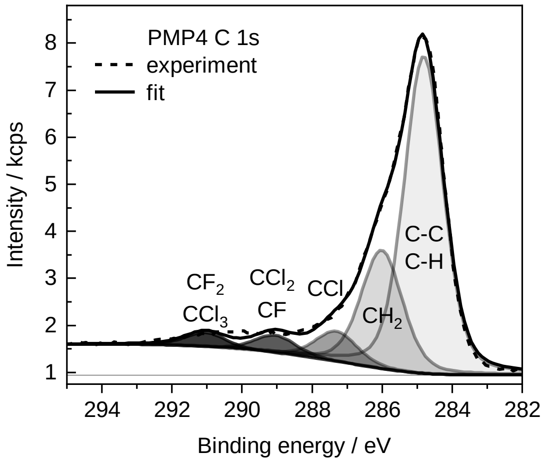

Supplementary Fig. 26: C 1s XPS survey spectrum of PMP4.

Supplementary Tab. 2: C 1s XPS analysis of PMP7 and PMP4.

C 1s	Peak name	Peak height / cps	Position / eV	FWHM / eV	Rel. Area / %
**PMP7**	C–C, C–H	3641.0	285.2	1.61	57.76
	CH_2, CCl	1374.1	286.4	2.08	28.05
	CF, CCl_2	402.0	289.4	2.37	9.33[a]
	CF_2, CCl_3	336.2	291.1	1.48	4.87[a]
**PMP4**	C–C, C–H	6659.8	284.8	1.33	62.19
	CH_2	2499.9	286.0	1.41	24.76
	CCl	627.9	287.3	1.40	6.36
	CCl_2, CF	327.6	289.0	1.40	3.72
	CF_2, CCl_3	280.3	291.1	1.40	2.95

[a] both relative areas were used for the calculation of the functionalisation degree of the graphitic material.

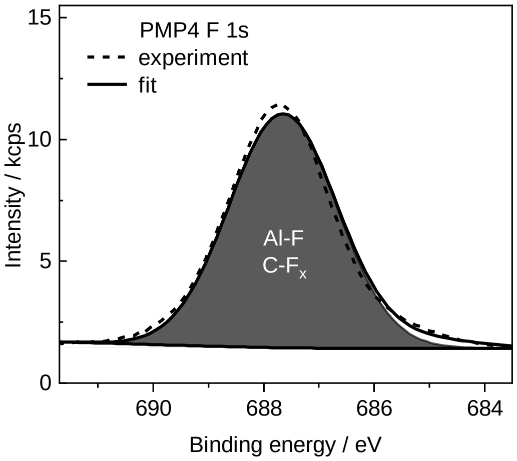

Supplementary Fig. 27: F 1s XPS survey spectrum of PMP4.

Supplementary Tab. 3: F 1p XPS analysis of PMP7 and PMP4.

F 1s	Peak name	Peak height / cps	Position / eV	FWHM / eV	Rel. Area / %
**PMP7**	CF_x [a] + Al–F	3233.3	687.1	3.2	100
**PMP4**	CF_x [a] + Al–F	9597.6	687.7	2.3	100

[a] x = 1, 2

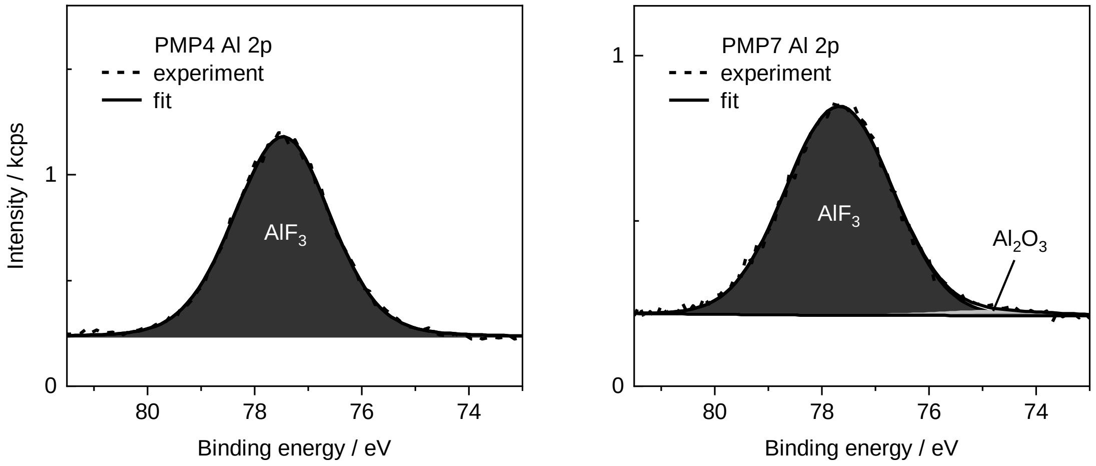

Supplementary Fig. 28: Al 2p XPS survey spectra of **PMP4** and **PMP7**.

Supplementary Tab. 4: Al 2p XPS analysis of **PMP7** and **PMP4**.

Al 2p	Peak name	Peak height / cps	Position / eV	FWHM / eV	Rel. Area / %
**PMP7**	Al–O	9.3	74.5	2.3	1.32
	Al–F	629.9	77.6	2.3	98.68
**PMP4**	Al–F	965.2	77.5	2.2	100.00

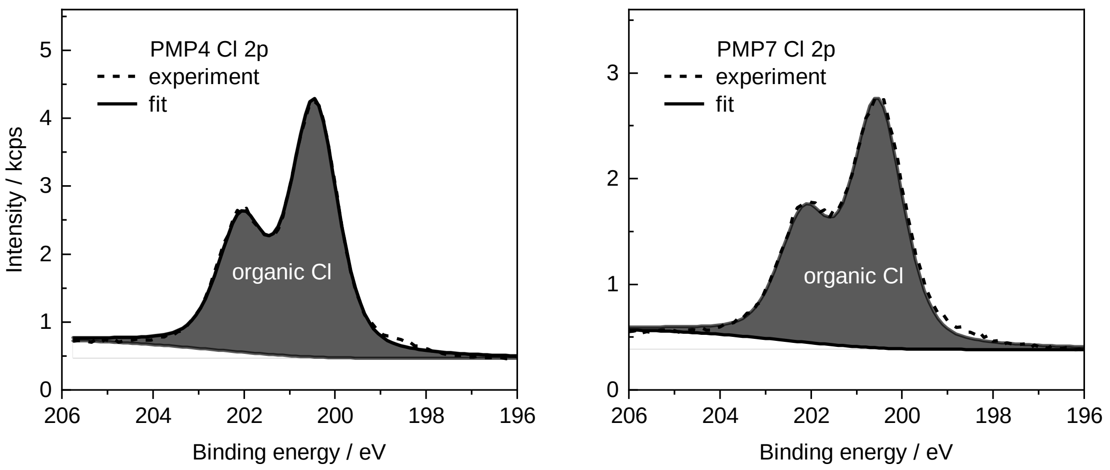

Supplementary Fig. 29: Cl 2p XPS survey spectra of **PMP4** and **PMP7**.

Supplementary Tab. 5: Cl 2p XPS analysis of **PMP7** and **PMP4**.

Cl 2p	Peak name	Peak height / cps	Position / eV	FWHM / eV	Rel. Area / %
**PMP7**	$p^{3}/_{2}$ CCl_x [a]	2290.1	200.5	1.3	66.67
	$p^{1}/_{2}$ CCl_x [a]	1145.1	202.1	1.3	33.33
**PMP4**	$p^{3}/_{2}$ CCl_x [a]	3806.1	200.4	1.4	66.67
	$p^{1}/_{2}$ CCl_x [a]	1903.0	202.0	1.4	33.33

[a] x = 1, 2

References

1. P. N. Grakovich, S. R. Allayarov, M. P. Confer, L. A. Kalinin, I. A. Frolov, T. N. Rudneva, L. F. Ivanov and D. A. Dixon, *J. Fluorine Chem.*, 2022, **255-256**, 109947.
2. W. R. Creasy and J. T. Brenna, *Chem. Phys.*, 1988, **126**, 453-468.
3. ISO 15472:2010, Surface chemical analysis — X-ray photoelectron spectrometers—calibration of energy scales, 2010
4. M. P. Seah, *J. Electron. Spectrosc. Relat. Phenom.*, 1995, **71**, 191-204.
5. R. Hesse and R. Denecke, *Surf. Interface Anal.*, 2011, **43**, 1514-1526.
